# Rectal roflumilast improves trinitrobenzenesulfonic acid-induced chronic colitis in rats

**DOI:** 10.1590/1414-431X2021e11877

**Published:** 2022-02-28

**Authors:** A. Shaikh-Omar, H.A. Murad, N.M. Alotaibi

**Affiliations:** 1 Department of Biology, Faculty of Science, King Abdulaziz University, Jeddah, Saudi Arabia; 2 Princess Dr. Najla Bint Saud Al Saud Center for Excellence Research in Biotechnology, King Abdulaziz University, Jeddah, Saudi Arabia; 3 Department of Pharmacology, Faculty of Medicine, Rabigh Campus, King Abdulaziz University, Jeddah, Saudi Arabia; 4 Department of Pharmacology, Faculty of Medicine, Ain Shams University, Cairo, Egypt

**Keywords:** Crohn's disease, Off-label, Rectal, Roflumilast, Phosphodiesterase IV

## Abstract

Roflumilast, a highly selective oral phosphodiesterase IV inhibitor, exerts anti-inflammatory and anti-fibrotic effects. Oral roflumilast causes gastrointestinal side effects, especially vomiting, which could be reduced by administering roflumilast via off-label routes. Inhaled roflumilast reportedly improved inflammatory and histopathological changes in asthmatic mice. The current study investigated the effects of oral and rectal roflumilast on trinitrobenzenesulfonic acid (TNBS)-induced chronic colitis in rats, an experimental model resembling human Crohn's disease. Five groups of rats (n=8) were used: normal control, TNBS-induced colitis, and three TNBS-treated colitic groups, which received oral sulfasalazine (500 mg·kg^-1^·day^-1^), oral roflumilast (5 mg·kg^-1^·day^-1^), or rectal roflumilast (5 mg·kg^-1^·day^-1^) for 15 days after colitis induction. Then, the following were assessed: the colitis activity score, tumor necrosis factor (TNF)-α, interleukin (IL)-2, and IL-6 serum levels, colonic length, and myeloperoxidase, malonaldehyde, and glutathione levels. Histological examinations employed H&E, Masson trichrome, and PAS stains in addition to immunostaining for KI-67 and TNF-α. The TNBS-induced colitis rats showed significant increases in disease activity scores, serum TNF-α, IL-2, and IL-6 levels, and colonic myeloperoxidase and malonaldehyde content. They also showed significant decreases in colonic length and glutathione levels in addition to histopathological and immunohistochemical changes. All the treatments significantly improved all these changes. Sulfasalazine provided the greatest improvement, followed by oral roflumilast, and then rectal roflumilast. In conclusion, both oral and rectal roflumilast partially improved TNBS-induced chronic colitis, suggesting the potential of roflumilast as an additional treatment for Crohn's disease.

## Introduction

The prevalence of Crohn's disease (CD), a predominant type of inflammatory bowel disease (IBD), has been increasing over the last decades. CD is a chronic, idiopathic, immunologically mediated disease that is triggered in a genetically predisposed person by multiple environmental factors (1). CD occurs as a patchy granuloma affecting any part of the gastrointestinal tract, especially the ileum and first part of the colon. It involves the entire bowel wall and manifests as abdominal masses and perianal lesions. Numerous anti-inflammatory or immunomodulatory treatments, including sulfasalazine and corticosteroids, are available for CD; however, they have many side effects and variable efficacy, especially in active CD (2). Thus, it is necessary to search for new medications for CD and, to be relevant to human CD, the animal model should be already established, chronic, and immune-mediated (3). Trinitrobenzene sulfonic acid (TNBS) induces an experimental model of colitis with a T helper 1 (Th1) immune pattern that is similar to human CD, but with certain limitations (4).

Phosphodiesterase IV (PDE4) inhibitors have anti-inflammatory effects that are rather similar to those of corticosteroids yet with the advantage of not interfering with the hypothalamo-pituitary-adrenal axis. Roflumilast, a highly selective oral PDE4 inhibitor with anti-inflammatory and anti-fibrotic effects, increases intracellular cyclic adenosine monophosphate and, hence, reduces the production of numerous inflammatory mediators such as tumor necrosis factor-α (TNF-α) and interleukins (ILs). It is the first licensed member of this class and has been approved by the FDA as an add-on treatment for chronic obstructive pulmonary disease. Moreover, in ovalbumin-induced asthmatic mice, inhaled roflumilast substantially improved inflammation and histopathological changes, as it decreased numbers of neutrophils, eosinophils, and macrophages in the bronchoalveolar lavage fluid. Thus, inhaled roflumilast could be a useful off-label treatment for neutrophilic and eosinophilic asthma (5). Oral roflumilast may cause weight loss, headache, nausea, emesis, and diarrhea. Its bioavailability is nearly 80%, and consuming food delays, but does not decrease, its absorption. Thus, taking roflumilast with food could improve its gastrointestinal side effects (6). To our knowledge, there is no rectal dosage form of roflumilast, but it can be administered rectally (7-9). If proven effective, rectal roflumilast administration for colitis will be useful because it avoids the side effects associated with the oral route. Furthermore, roflumilast could have additional benefits for CD patients. It exerts anti-diarrheal and anti-spasmodic effects by inhibiting PDE4 and voltage-gated Ca^++^ channels at low doses (10). Moreover, in 1,2-dimethylhydrazine-induced preneoplastic colon damage in rats, subcutaneous roflumilast improved colonic damage, decreasing oxidative stress and inflammatory markers, stabilizing hemodynamic imbalances, and restoring normal colonic mucosa architecture (11).

Taken together, we hypothesized that treatment with rectal roflumilast will significantly improve TNBS-induced chronic colitis in rats. The current study tested the effects of oral and rectal roflumilast on TNBS-induced chronic colitis in rats, an experimental model that resembles human CD. If proven effective, roflumilast could have potential as an add-on off-label treatment for CD.

## Materials and Methods

### Animals

This study was approved by the Ethics Committee at King Abdulaziz University (No. 499/19) and adhered to the international guidance for use of laboratory animals. Chemicals and drugs were purchased from Sigma-Aldrich Corp. (USA) unless mentioned otherwise. Female Sprague-Dawley rats (180-200 g) were housed in cages at 22°C with a 12-h light/dark cycle. Rats were acclimatized for 7 days before starting the procedures. Food and water were available *ad libitum*.

### Induction of colitis and treatment groups

Chronic colitis was induced by weekly rectal injections of increasing doses of TNBS over 6 weeks (15, 30, 45, 60, 60, and 60 mg from day 0 to day 35), as previously described (4,12). Briefly, after food deprivation for 24 h, rats were anesthetized with an intraperitoneal injection of 1% isoflurane. TNBS was dissolved in ethanol (50%) and instilled into the colon using a cannula (0.25 mL), and then the rats were held upside down for 3 min to prevent escape of the intracolonic TNBS. Roflumilast was dissolved in dimethyl sulfoxide and diluted in 0.5% carboxymethyl cellulose sodium to a final concentration of 5.0 mg·kg^-1^·day^-1^ as previously described (13). Five groups of rats (n=8) were established: normal control (NC group), TNBS-treated (TNBS group, positive control), and three TNBS-treated groups that received oral sulfasalazine (SS group, 500 mg/kg) (14), oral roflumilast (by gastric gavage, OR group, 5 mg/kg), or rectal roflumilast (RR group, 5 mg/kg). Treatments were administered once daily in a volume of 200 µL for 15 days starting 48 h after the induction of colitis (from day 37) (15), and the control rats received equal amounts of vehicle. At the end of the treatment period, the rats were evaluated with the colitis activity score. To detect the effects of the treatments on systemic inflammatory processes, blood was collected for measurements of the proinflammatory mediators TNF-α, IL-2, and IL-6. At the end of the 15-day treatment period, the rats were sacrificed by cervical dislocation, and the colon was isolated, cleaned of feces, and measured for weight and length. Colonic myeloperoxidase (MPO) activity and glutathione (GSH) and malonaldehyde (MDA) levels were measured. The colon was examined macroscopically for gross changes and microscopically in sections stained with hematoxylin and eosin (H&E), Masson trichrome, and Periodic Acid Schiff (PAS) stains as well as KI-67 and TNF-α immunostaining.

### Disease activity score

The colitis activity index was determined to evaluate the activity of intestinal inflammation as previously described with a slight modification (16). The combined score was calculated based on the following parameters: i) weight loss (0: no loss; 1: 1-15%; 2: >15%); ii) blood in feces (0: no blood; 1: traces of blood (≤50% of surface); 2: gross bleeding (>50% of surface); and iii) consistency of feces (0: ordinary; 1: loose stools; 2: diarrhea).

### Colonic macroscopic examination

After the rats were sacrificed, their colon was excised, and its length was measured. The appearance of the colon regarding edema, ulceration, and necrosis was evaluated (17).

### Serum measurements

Blood was collected through cardiac puncture. The serum was obtained and stored at −80°C until analysis. The measurements were done using ELISA kits for TNF-α, IL-2, and IL-6 (Innovative Research, USA), according to the manufacturer's guidelines.

### Colonic measurements

The levels of MPO (a marker of neutrophil activity), GSH (one of the most important cellular antioxidants), and MDA (an indicator of lipid peroxidation) in colonic tissue homogenates were measured using the commercially available kits as previously described: MPO ELISA kit (Innovative Research) (18), GSH colorimetric assay kit (Innovative Research) (19), and MDA ELISA kit (Neobio Lab Comp., USA) (20).

### Histopathological examination

The colonic segments were fixed in 10% buffered formalin and embedded in paraffin. Afterwards, 3-5-µm-thick sections were made and stained with H&E, Masson's trichrome for collagen, and PAS for lymphocytes and mucopolysaccharides. The severity of the lesions was evaluated by a blinded histopathologist as normal, mild, moderate, or marked injury (21).

### Immunohistochemical examination

The colonic sections were immunohistochemically stained with KI-67 and TNF-α. For KI-67 immunostaining, the avidin biotin peroxidase method was used as previously described (22). Briefly, the colonic sections were mounted on charged slides, deparaffinized, and washed in buffered phosphate-buffered saline (PBS) (pH 7.2) for 5 min. The sections were incubated with antisera with the specific KI-67 primary antibody at 1:80 dilution (Dako, M7240, USA) in a humidified chamber at room temperature overnight. Afterwards, the slides were washed in PBS, incubated with a horseradish peroxidase polymer for 15 min at room temperature, washed again in PBS, and incubated in diaminobenzidine as chromogen. The sections were then stained with Mayer's hematoxylin, dehydrated, mounted, and examined by light microscopy. For TNF-α immunostaining, the avidin biotin peroxidase method was used as previously described (23). The colonic sections were deparaffinized, rehydrated, and heated in a microwave oven in 0.01 M citrate buffer (pH 6.0) for 30 min. The sections were incubated at 4°C overnight with anti-TNF (rabbit polyclonal IgG, Santa Cruz Biotechnology Inc., USA). This primary antibody was detected using avidin-biotin peroxidase detection solution (Dako), and the signal was visualized using diaminobenzidine. Slides were counterstained with Harris's hematoxylin, dehydrated, and then examined by light microscopy.

### Statistical analysis

Data are reported as means±SE and were analyzed using SPSS version 22 (IBM, USA). One-way ANOVA and Tukey's *post hoc* tests were used to test differences among groups. P<0.05 was considered statistically significant.

## Results

### Disease activity score

**Figure 1:**
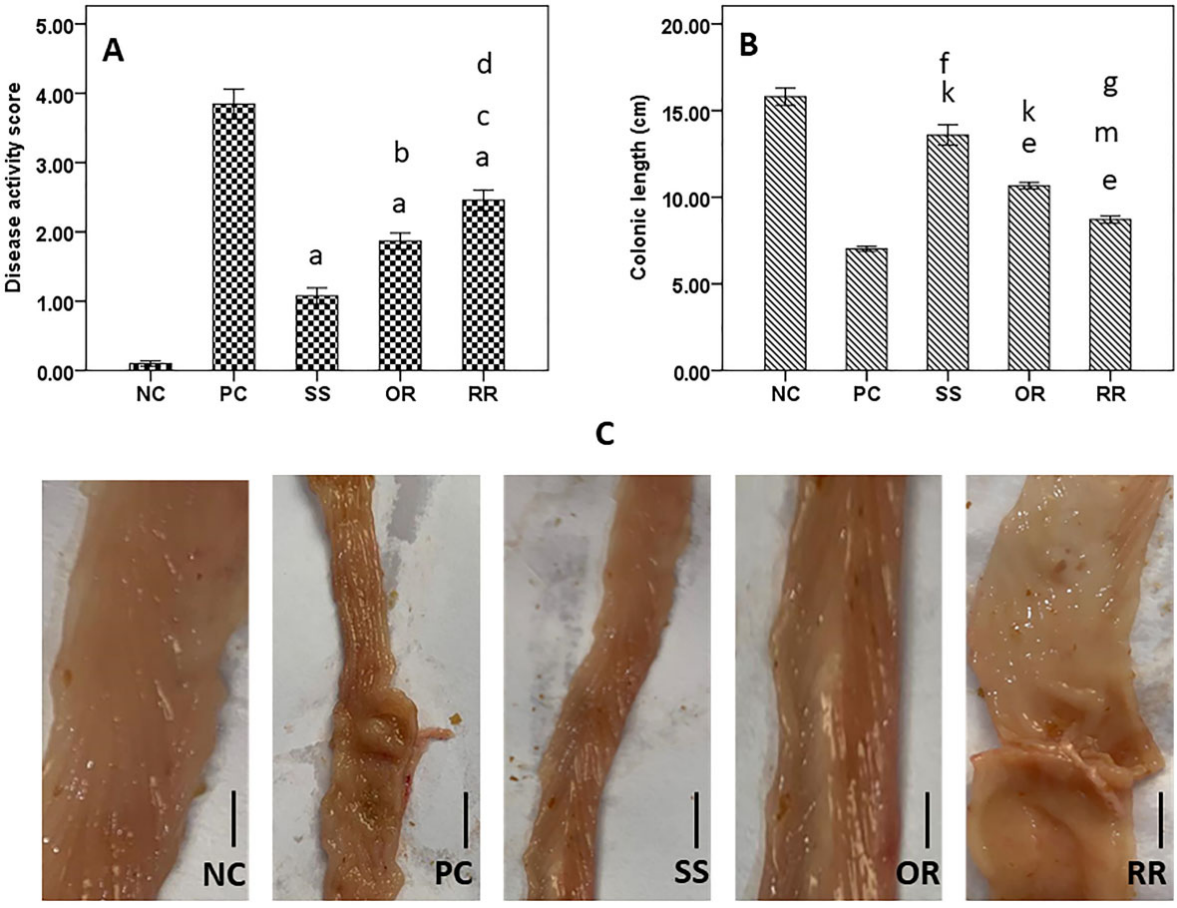
The effects of roflumilast in trinitrobenzenesulfonic acid (TNBS)-induced colitis in rats. **A**, Disease activity score: ^a^P<0.001 SS & OR & RR *vs* NC and PC, ^b^P<0.01 OR *vs* SS (P=0.003), ^c^P<0.001 RR *vs* SS, ^d^P<0.05 RR *vs* OR (P=0.037). **B**, Colon length: ^e^P<0.001 OR & RR *vs* NC, ^f^P<0.01 SS *vs* NC (=0.001), ^g^P<0.05 RR *vs* PC (=0.023), ^k^P<0.001 SS & OR *vs* PC, ^m^P<0.01 RR *vs* OR (=0.006). **C**, Colonic macroscopic appearance. The scale bar indicates 1 cm: The NC group shows a normal appearance of the colon. The PC group shows severe colonic injury characterized by inflammation, mucosal edema, and thickening of the bowel wall. The SS group shows marked improvement, the OR group shows moderate to marked improvement, and the RR group shows mild improvement. Data in (**A**) and (**B**) are reported as means±SE; one-way ANOVA and Tukey's *post hoc* test. NC: normal control; PC: positive control; SS: sulfasalazine; OR: oral roflumilast; RR: rectal roflumilast.

The TNBS-induced colitis rats showed an increased disease activity score compared with NC. All treatments significantly decreased this score. Sulfasalazine exerted the greatest decrease, and oral roflumilast decreased the score more than rectal roflumilast (Figure 1A).

### Colonic length and macroscopic appearance

TNBS-induced colitis rats showed shorter colonic length compared with NC rats, and all treatments significantly preserved the length. Sulfasalazine exerted the greatest effect, and oral roflumilast caused more improvement than rectal roflumilast (Figure 1B). The appearance of the colon regarding inflammation, mucosal edema, erosions, and necrosis was evaluated. TNBS-induced colitis rats showed severe colonic lesions characterized by damaged mucosa and thickened bowel walls. The sulfasalazine group showed marked improvement, the oral roflumilast group showed moderate to marked improvement, and the rectal roflumilast group showed mild improvement (Figure 1C).

### Serum measurements

TNBS-induced colitis rats showed significantly increased serum levels of TNF-α, IL-2, and IL-6. All treatments significantly reversed these TNBS-induced changes. Sulfasalazine exerted the greatest decrease, and oral roflumilast decreased the levels more than rectal roflumilast (Table 1).

**Table 1:** The effects of roflumilast on serum levels of TNF-α, IL-2, and IL-6 in rats with trinitrobenzenesulfonic acid (TNBS)-induced colitis (n=8).

	NC	PC	SS	OR	RR
TNF-α (pg/mL)	17.04±0.67	91.18±4.14	28.93±0.5^b,d^	38.87±2.33^a,b,f^	79.37±1.88^a,c,e^
IL-2 (pg/mL)	196.10±3.19	487.00±9.36	257.97±7.72^g^	302.75±11.32^g,h,i^	363.87±12.73^g^
IL-6 (pg/mL)	10.57±0.42	47.04±1.98	16.14±1.13^k,m^	21.85±1.00^j,k,l^	28.93±0.50^j,k^

Data are reported as means±SE; one-way ANOVA and Tukey's post hoc test. NC: normal control; PC: positive control; SS: sulfasalazine; OR: oral roflumilast; RR: rectal roflumilast. Tumor necrosis factor-α (TNF-α): ^a^P<0.001 OR & RR *vs* NC; ^b^P<0.001 SS & OR *vs* PC; ^c^P<0.001 RR *vs* SS & OR; ^d^P<0.01 SS *vs* NC (=0.008); ^e^P<0.01 RR *vs* PC (=0.008); ^f^P<0.05 OR *vs* SS (=0.034). Interleukin-2 (IL-2): ^g^P<0.001 all treatments *vs* NC & PC; ^h^P<0.01 OR *vs* RR (=0.001); ^i^P<0.05 OR *vs* SS (=0.016). Interleukin-6 (IL-6): ^j^P<0.001 OR & RR *vs* NC; ^k^P<0.001 all treatments *vs* PC; ^l^P<0.01 OR *vs* RR (=0.001); ^m^P<0.05 SS *vs* NC & OR (=0.013, 0.010).

### Colonic measurements

TNBS-induced colitis rats showed significantly increased levels of MPO and MDA and significantly decreased GSH levels in the colon homogenates. All treatments significantly reversed these TNBS-induced changes. Sulfasalazine exerted the greatest changes, and oral roflumilast caused more changes than rectal roflumilast (Table 2).

**Table 2:** The effects of roflumilast on colonic levels of MPO, MDA, and GSH in rats with trinitrobenzenesulfonic acid (TNBS)-induced colitis (n=8).

	NC	PC	SS	OR	RR
MPO activity (μ/g)	11.29±0.85	50.70±1.05	18.38±0.87^a^	23.89±0.92^a,b,c^	28.17±1.00^a^
MDA (mmol/mg)	19.31±0.66	81.40±2.13	28.93±0.50^e^	36.68±1.98^d,e,f,g^	47.04±1.98^d,e^
GSH (mmol/mg)	19.60±0.79	6.86±0.22	14.73±0.49^h,i^	11.94±0.32^h,i,k^	9.24±0.28^h,j^

Data are reported as means±SE; one-way ANOVA and Tukey's post hoc test. NC: normal control; PC: positive control; SS: sulfasalazine; OR: oral roflumilast; RR: rectal roflumilast. Myeloperoxidase (MPO): ^a^P<0.001 all treatments *vs* NC & PC; ^b^P<0.01 OR *vs* SS (=0.002); ^c^P<0.05 OR *vs* RR (=0.022). Malonaldehyde (MDA): ^d^P<0.001 OR & RR *vs* NC; ^e^P<0.001 all treatments *vs* PC; ^f^P<0.01 OR *vs* RR (=0.001); ^g^P<0.05 OR *vs* SS (=0.014). Glutathione (GSH): ^h^P<0.001 all treatments *vs* NC; ^i^P<0.001 SS & OR *vs* PC; ^j^P<0.01 RR *vs* PC (=0.008); ^k^P<0.01, OR *vs* SS & RR (=0.001, 0.002).

### Histopathological examination

**Figure 2:**
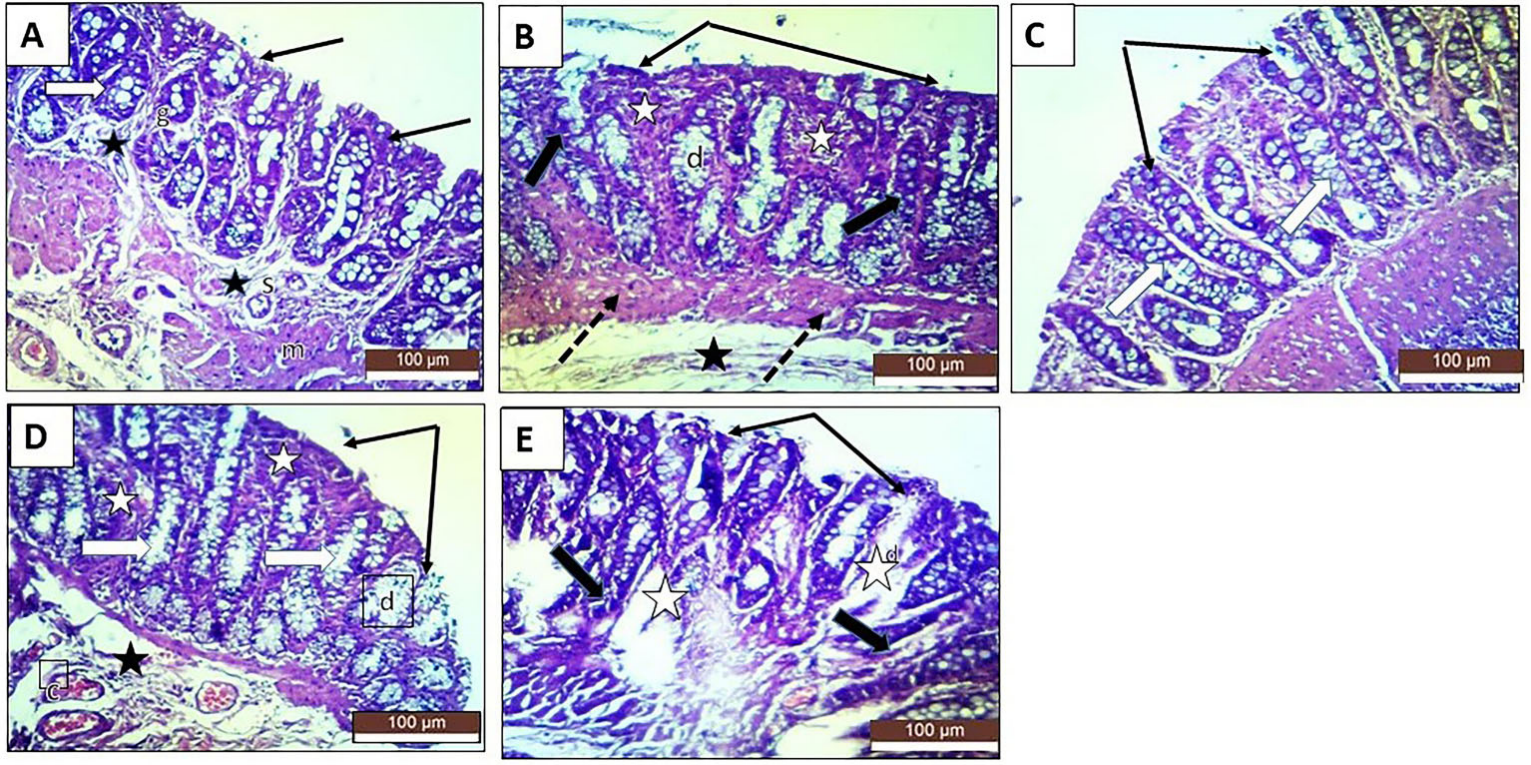
Photomicrographs of rat colonic sections stained with H&E (×20, scale bar 100 μm). **A**, The normal control group shows normal mucosal layers, intact surface epithelium (black arrows), normally shaped crypts with numerous goblet cells (white arrow), and narrow connective tissue submucosa (black stars). Tunica submucosa (s) consisting of loose connective tissue rich in blood vessels and lymph vessels, and muscularis mucosae (m) consisting of inner circular and outer longitudinal smooth muscle fibers are seen. **B**, The positive control group shows loss of surface crypt epithelium (black arrows) and destruction of crypts that appear darker with less goblet cells (d). The regions of the lost crypts are replaced by inflammatory infiltrate and fibrous tissue (white stars). Thickened fibrosed muscularis muscle and widened submucosa due to edema (black star) are seen. **C**, The sulfasalazine group shows marked improvement with nearly normal appearance of colonic surface (thin black arrows) and crypts (thick white arrows) with few desquamated cells, especially colonocytes. **D**, The oral roflumilast group shows moderate improvement with focal loss of surface crypt cells (black arrows) and some lost crypts (white stars). Other crypts appear normal with numerous goblet cells (white arrows). The submucosa is somewhat edematous (black star) with congested blood vessels (c). The colonic wall displays thickening with patches of fibrosis and epithelial desquamation (d). **E**, The rectal roflumilast group shows mild improvement with apparent thickening of the colonic mucosa with numerous patches of crypt loss (white stars) and dark degenerated surface cells (thin black arrows). The crypts appear deformed, degenerated, and dark, with few goblet cells (thick black arrows). Desquamated colonic cells (d), especially the cells lining the crypts, and numerous congested blood vessels in the propria submucosa are seen.

**Figure 3:**
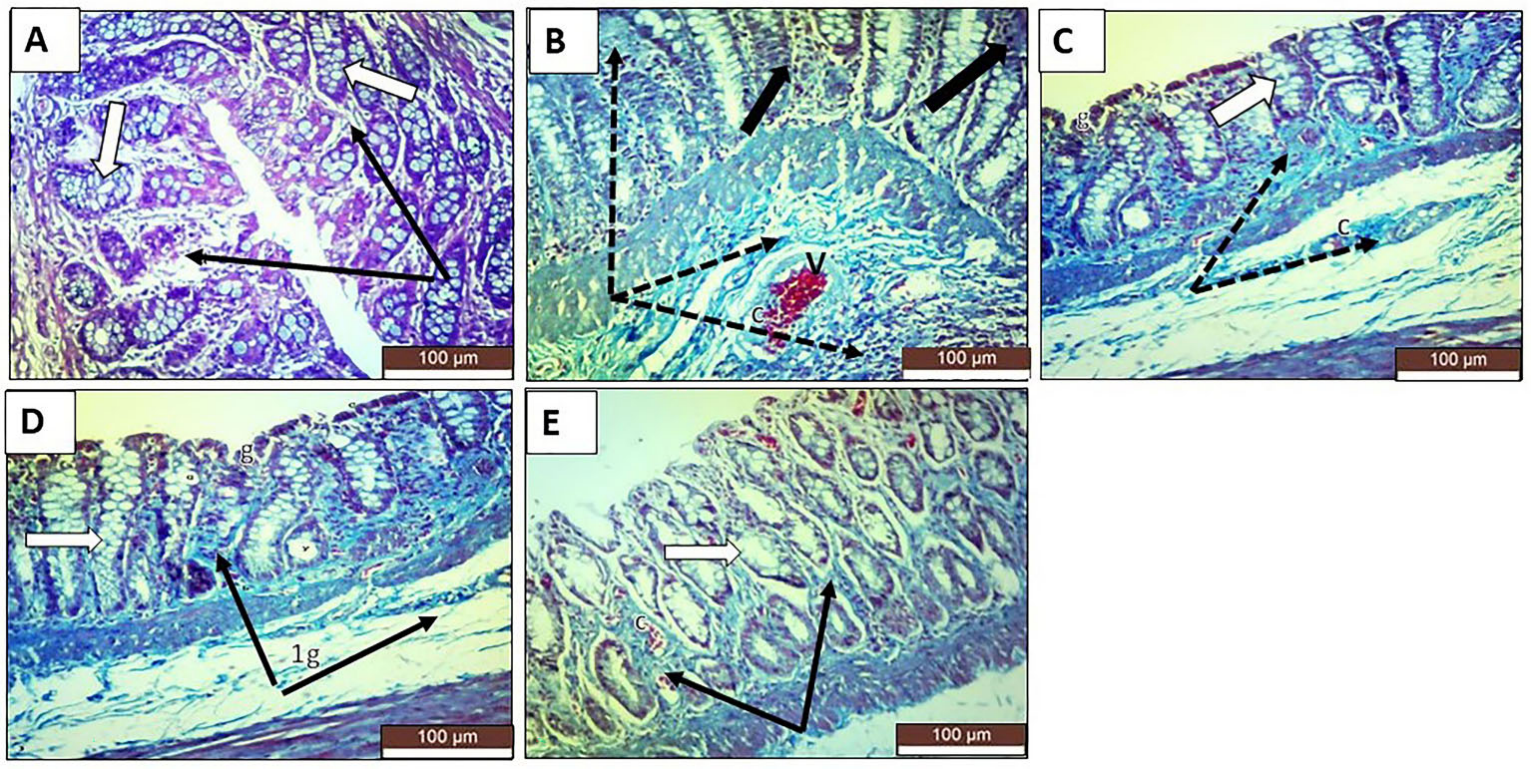
Photomicrographs of rat colonic sections stained with Masson trichrome (×20, scale bar 100 μm). **A**, The normal control group shows colonic mucosa with normal crypts and goblet cells (white arrows) separated by scanty loose, blue-stained collagen (thin black arrows). **B**, The positive control group shows a marked increase in collagen deposition (dotted black arrows) replacing the degenerated crypts (thick black arrows) and around the congested and thickened blood vessels (c) in the submucosa. **C**, The sulfasalazine group shows a marked decrease in collagen (dotted black arrows) deposition between crypts (white arrow). Some crypts of Lieberkühn showed ulceration and leucocyte accumulation. Some congested blood vessels (c) in the propria submucosa and numerous goblet cells (g) are seen. **D**, The oral roflumilast group shows a moderate decrease in collagen fibers (black arrows) between colonic crypts (white arrow) and in submucosa. Numerous goblet cells (g) and some congested blood vessels are seen between the crypts of Lieberkühn. **E**, The rectal roflumilast group shows a mild decrease in collagen deposition (black arrows) between colonic crypts (white arrow), ulcerated crypts of Lieberkühn, and congested blood vessels (c) with leucocyte accumulation.

**Figure 4:**
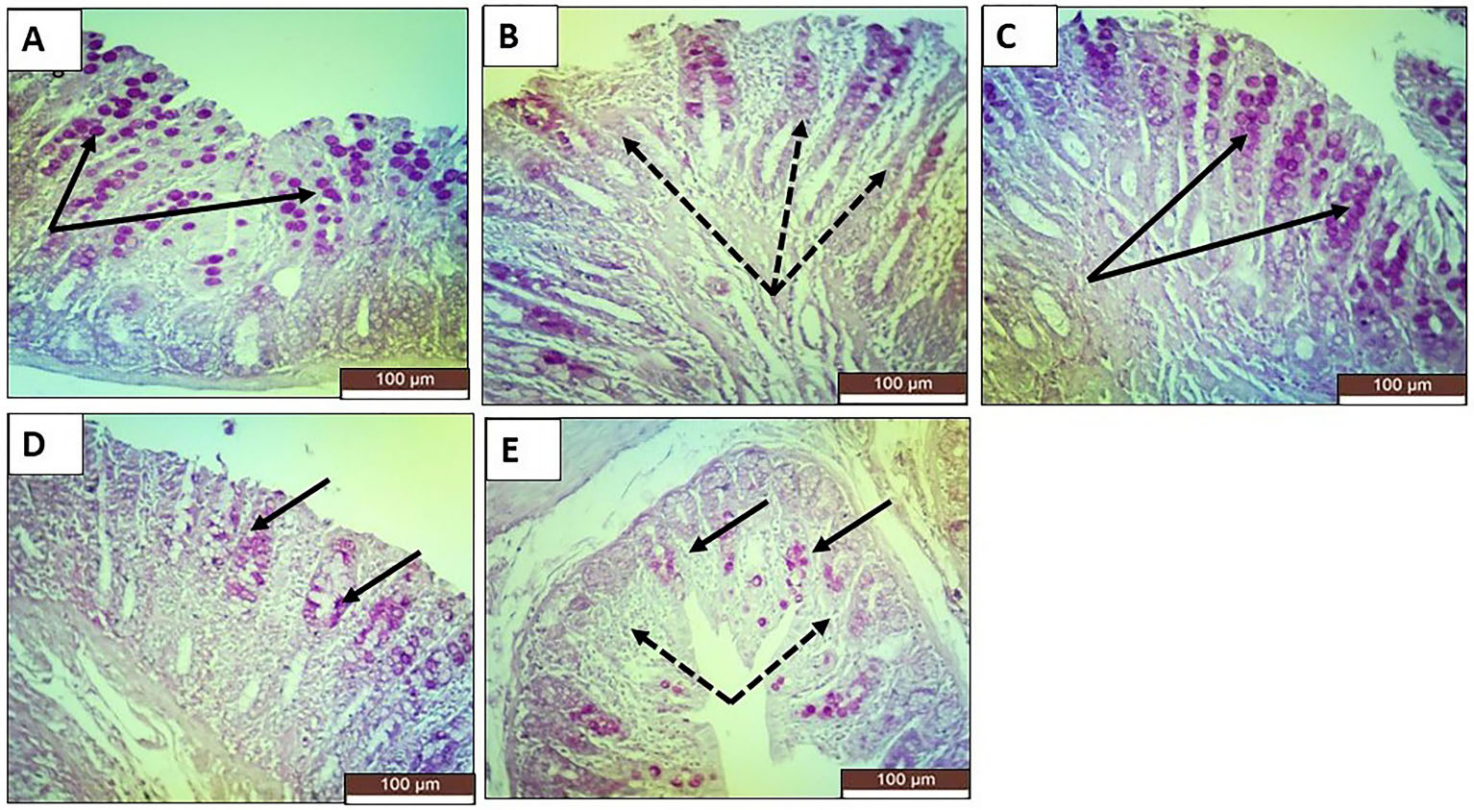
Photomicrographs of rat colonic sections stained with PAS (×20, scale bar 100 μm). **A**, The normal control group shows colonic mucosa with normal crypts and numerous PAS-stained goblet cells (black arrows). **B**, The positive control group shows a marked decrease or complete loss of PAS-stained goblet cells (dotted black arrows). **C**, The sulfasalazine group shows a nearly normal population of PAS-stained goblet cells (black arrows). **D**, The oral roflumilast group shows a moderate population of PAS-stained goblet cells (black arrows). **E**, The rectal roflumilast group shows a small population of PAS-stained goblet cells (black arrows) indicating faint positive PAS reactions with patches of decrease or complete loss (dotted black arrows).

In the colonic sections stained with H&E, the TNBS-induced colitis rats exhibited destroyed glands, marked inflammatory infiltration, focal necrosis of mucosa and submucosa, loss of epithelial lining, and diffuse submucosal edema (Figure 2). Masson trichrome staining for collagen and collagen fibers revealed that the TNBS group showed numerous congested blood vessels in the propria submucosa with edema around the connective tissue fibers and blood vessels. Lymphocytes accumulated in the propria submucosa, and some large nodules were located in the mucosa-submucosa (Figure 3). PAS staining for mucopolysaccharides revealed that the TNBS group showed numerous goblet cells with positive PAS reactions that were prominent between the epithelial lining of the crypts. Some crypts of Lieberkühn showed ulceration and leucocyte accumulation (Figure 4). All treatments exerted significant prophylactic effects, preventing the inflammatory changes to occur and interfering with the induction of chronic colitis by TNBS. Sulfasalazine exerted the greatest improvement, and oral roflumilast caused more improvement than rectal roflumilast.

### Immunohistochemical results

**Figure 5:**
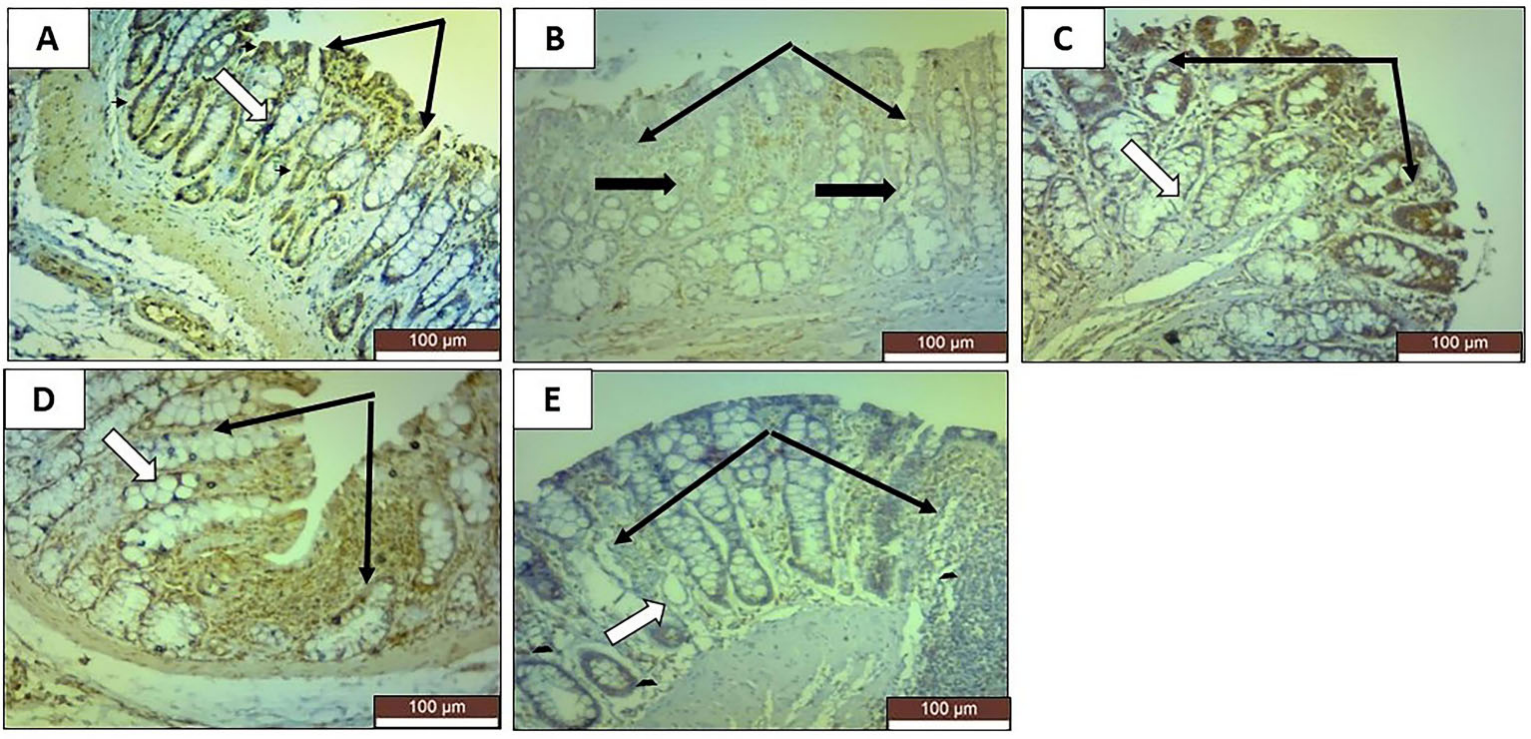
Photomicrographs of rat colonic sections stained for KI-67 immunoexpression in the mucosa (×20, scale bar 100 μm). **A**, The normal control group shows strong positive reactions indicating extensive immunoexpression in crypt cells (white arrow) and surface cells (black arrows). **B**, The positive control group shows very faint positive reactions indicating very mild immunoexpression in crypt cells (thick black arrows) and surface cells (thin black arrows). **C**, The sulfasalazine group shows moderately positive reactions indicating moderate immunoexpression in crypt cells (white arrow) and surface cells (black arrows). **D**, The oral roflumilast group shows mildly positive reactions indicating weak immunoexpression in crypt cells (white arrow) and surface cells (black arrows). **E**, The rectal roflumilast group shows very mild positive reactions indicating very weak immunoexpression in crypt cells (white arrow) and surface cells (black arrows).

**Figure 6:**
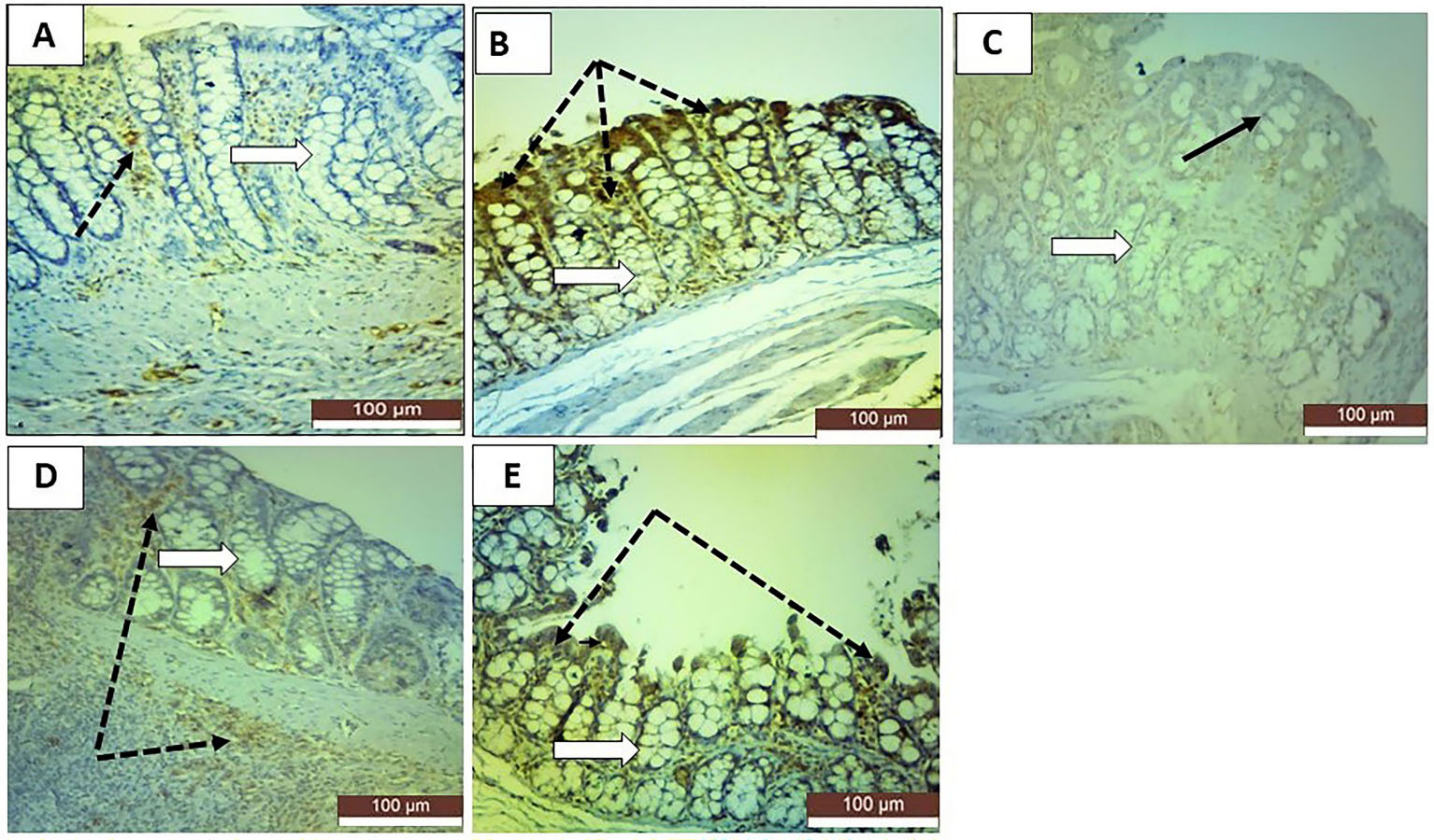
Photomicrographs of rat colonic sections stained for TNF-α immunoexpression in the mucosa (×20, scale bar 100 μm). **A**, The normal control group shows negative reactions in the crypt cells and goblet cells (white arrow), except for a few cells in the connective tissue lamina (dotted black arrow). **B**, The positive control group shows highly positive reactions in the degenerated crypt cells (white arrow) and inflammatory cells between the crypts (dotted black arrows). **C**, The sulfasalazine group shows very faint positive reactions in the surface cells (black arrow) and crypt cells (white arrow). **D**, The oral roflumilast group shows moderately positive reactions in the surface cells (dotted black arrows) and crypt cells (white arrow). **E**, The rectal roflumilast group shows strong positive reactions in crypt cells (white arrow) and degenerated cells (dotted black arrows).

KI-67 staining revealed very faint positive reactions in the colonic sections of TNBS-induced colitis rats (Figure 5). TNF-α staining revealed strong positive reactions in the TNBS group (Figure 6). Both stains revealed that all the treatments reversed these TNBS-induced changes to different degrees. Sulfasalazine exerted the greatest improvement, and oral roflumilast caused more improvement than rectal roflumilast.

## Discussion

The chronic TNBS-induced colitis model is used to induce several CD-like features, such as transmural inflammation, Th1 immune pattern colitis, and fibrosis (4,12). A systematic review concluded that the chronic TNBS-induced colitis preclinical model can be acquired with several TNBS administrations and is most commonly characterized by the concentrations of inflammatory biomarkers, e.g., TNF-α and IL-6 (24). Inflammatory cytokines are crucial in immune responses, and their increased levels are related to the pathogenesis of IBD pathogenesis. The advantage of the chronic compared with the acute model is that the acute model only causes injury to the epithelium, leading to self-limiting inflammation rather than chronic disease (25). Additionally, in chronic TNBS-induced colitis, the parameters for histological evaluation of colonic sections stained with H&E included epithelial damage and inflammatory infiltration. TNBS-induced chronic colitis was characterized by submucosal edema, mucosal damage, and severe inflammatory infiltration. Cytokines, including IL-2, IL-6, and TNF-α, were measured in blood for cytokine profiling and to identify which cytokines best discriminate experimental colitis from controls (26). The histopathological evaluation of TNBS-induced chronic colitis included severity of inflammation and mucosal epithelial lesion; generally, diffuse transmural necrosis with hemorrhage was observed (24,27).

PDE4 is an intracellular enzyme that increases the production of inflammatory mediators and reduces the production of anti-inflammatory mediators. As such, it is implicated in the pathogenesis of many inflammatory diseases. PDE4 inactivates cyclic adenosine monophosphate and is the main PDE isoenzyme in mononuclear inflammatory cells, the principal source of TNF-α (6). It was reported that the increase in TNF-α plays a crucial role in the pathogenesis of IBD. IL-2 is another common cellular inflammatory factor. In inflammatory conditions, TNF-α and IL-2 levels rapidly increase and thus activate white blood cells, promote the migration of inflammatory cells, and expand the inflammatory response (28). Inhibition of PDE4 leads to widespread anti-inflammatory effects including decreased TNF-α levels. Thus, specific inhibition of PDE4 could be effective in treating numerous chronic inflammatory disorders (29). Interestingly, roflumilast is clinically effective at relatively small doses compared with the other PDE4 inhibitors. It improved episodic memory in subjects with minimal cognitive impairment at a low non-emetic dose with a plasma level nearly five-fold lower than the dose for chronic obstructive pulmonary disease patients. Using such low doses minimizes typical side effects of PDE4 inhibitors such as vomiting (30). Roflumilast showed a more potent anti-inflammatory activity in both animals and humans and was better tolerated than the preceding PDE4 inhibitors, e.g., rolipram and cilomilast (31).

The results of the current study are in agreement with a previous study that revealed that oral roflumilast (1 or 5 mg·kg^-1^·day^-1^) dose-dependently improved the disease clinical score (weight loss, stool consistency, and hemorrhage), colonic length, and colonic TNF-α production in mice with dextran sulphate sodium (DSS)-induced colitis (13). However, the histological score was not improved in the previous study (13). In addition, roflumilast showed potential anti-inflammatory effects in DSS-induced ulcerative colitis in male Wistar rats. Colitis was defined by assessing weight loss, colonic length, histological score, TNF-α levels, nitric oxide levels, cyclic adenosine monophosphate levels, MPO activity, and inducible nitric oxide synthase gene expression in colonic tissue. Roflumilast (5 mg/kg) diminished the severity of colitis, as it preserved colon length, decreased weight loss, and improved histological scores compared with the DSS group. It also decreased colon concentrations of TNF-α and NO, MPO activity, and inducible nitric oxide synthase gene expression. The effects of roflumilast were comparable to those exerted by sulfasalazine (32).

In the current study, TNBS-induced chronic colitis showed severe colonic injury characterized macroscopically by shortened colonic length, inflammation, mucosal edema, and thickening of the bowel wall. It was reported that TNBS-chronic colitis can be evaluated macroscopically with colon weight, length, wall thickness, and signs of inflammation, including hyperemia and mucosal edema (24). Colon length is considered an indirect measure of tissue integrity and severity of inflammation. TNBS-induced colitis decreases colon length, increases bowel thickness, and causes ulceration and hyperemia (33). Our results were in agreement with another study that reported that roflumilast partially reverses the TNBS-induced reduction in colon length at 1 and 5 mg·kg^-1^·day^-1^ and decreases the elevated colonic concentration of TNF-α (13).

In the current study, we investigated the effects of rectal roflumilast on TNBS-induced chronic colitis. Our results showed that rectal roflumilast reversed the TNBS-induced colitic changes, suggesting its local anti-inflammatory effects. A previous study on colitis-induced rats reported that oral roflumilast has local anti-inflammatory effects. Oral roflumilast dose-dependently improved the clinical score of colitis, reduced the colonic shortening, and decreased local TNFα expression in colonic tissue; however, this improvement was not correlated with a decreased histological score (13). Additionally, in a clinical trial, roflumilast cream applied topically once daily to affected areas of psoriasis was superior to vehicle cream in causing an almost clear state at 6 weeks. Thus, it seems that topical roflumilast has the potential to help existing therapies in many inflammatory skin diseases (34).

In the current study, the colonic sections of the TNBS-induced colitis group showed destroyed glands, marked inflammatory infiltration, numerous congested blood vessels with edema, loss of goblet cells, and ulceration in crypts. Roflumilast improved the TNBS-induced histopathological and immunohistochemical changes in rats. This is in agreement with a previous study that showed a close relationship between crypt injuries and clinical colitis activity (35), but contradicts another study that reported that roflumilast does not significantly change the histological score (13). The antigen KI-67 is a nuclear protein that is considered a marker of cellular proliferation. KI-67 is present during the active phases of the cell cycle and absent during the resting phase (36). In DSS-induced ulcerative colitis, epithelial apoptosis increased approximately five-fold, and the number of mitotic cells decreased by almost a half compared with the control group. The KI-67 immunohistochemical examination showed that crypt cells with cell cycle arrest at the resting stage increased nearly two-fold compared with the control group, indicating reduced proliferation. This might disrupt the epithelial barrier mechanism, facilitating mucosal invasion by intraluminal microorganisms (22). In TNBS-induced colitis, decreased KI-67 expression in colon epithelium indicates a smaller number of epithelial cells undergoing proliferation compared with the normal control. The number of KI-67-positive epithelial cells increased with treatment, implying regeneration of the injured epithelium (37). In TNBS-induced colitis, the increased TNF-α immunoreactivity in colonic tissue was significantly reduced by anti-inflammatory treatments in rats (38,39).

Limitations of the current study include the measurements of cytokine levels in the blood. Although the aim was cytokine profiling and to identify which cytokines best discriminate experimental colitis from controls, using Western blot or ELISA to measure these cytokines in colonic tissue would have been more useful. Furthermore, some parameters of chronic inflammation, such as transforming growth factor-β or fibronectin, were not evaluated, and the TNBS-induced colitis model itself cannot mimic the relapsing phase of CD (40).

In conclusion, both oral and rectal roflumilast partially improved TNBS-induced chronic colitis, an experimental model resembling human CD, indicating local anti-inflammatory effects. Oral roflumilast caused more improvement than rectal roflumilast but was generally inferior to sulfasalazine, the standard treatment of CD. Nevertheless, our findings suggested that roflumilast could be used either via the oral or rectal route as an add-on off-label treatment for CD. Further studies should explore the additional mechanisms of action of roflumilast in IBD.
